# Portuguese cross-cultural adaptation and validation of the hospital survey on patient safety culture 2.0

**DOI:** 10.1186/s12913-025-12960-x

**Published:** 2025-06-05

**Authors:** Elsa Freitas, Carina Silva, Margarida Eiras

**Affiliations:** 1 Santarém District Hospital, Santarém, Portugal; 2 Centro de Estatística e Aplicações, Universidade de Lisboa, Lisbon, Portugal; 3 School of Health Technologies of Lisbon, Polytechnic Institute of Lisbon, Lisbon, Portugal

**Keywords:** Patient safety culture, Validity, Reliability, HSOPSC 2.0

## Abstract

**Background:**

As patient safety continues to be a global priority, it is crucial to emphasize the assessment and development of Patient Safety Culture [PSC] to advance healthcare quality and safety initiatives worldwide. This study focused on the assessment of PSC in Portuguese hospitals, specifically through the translation and cultural adaptation of the Hospital Survey on Patient Safety Culture 2.0 [HSOPSC 2.0] for the Portuguese context.

**Methods:**

The study followed a two-phase design, encompassing the translation, cultural adaptation, and psychometric evaluation of HSOPSC 2.0. A total of 2,604 fully completed questionnaires were collected. The sample consisted of seven public hospitals from different regions of Portugal, ensuring broad representation within the Portuguese National Health System. Translation process include four stages: forward translation, back translation, expert panel review, and pre-testing. Instrument reliability was assessed using Cronbach’s alpha coefficients and Exploratory Factor Analysis (EFA). Construct validity was evaluated through Confirmatory Factor Analysis (CFA), while convergent and discriminant validity were examined using Average Variance Extracted (AVE) and Pearson’s correlation coefficients, respectively.

**Results:**

The Portuguese translated version of the HSOPSC 2.0 [PT-HSOPSC 2.0] demonstrated good internal consistency, with Cronbach’s alpha values ranging from 0.63 to 0.88, and factor loadings above 0.80 indicating strong factor reliability. CFA results supported the adequacy of the model fit: χ²/df = 4.64, *p* < 0.01; RMSEA = 0.05; CFI = 0.93; GFI = 0.90; TLI = 0.91; PCFI = 0.78; PGFI = 0.71. The instrument demonstrated good convergent validity, with AVE values at or above 0.50. Strengths included “Teamwork” and “Management Support for Patient Safety”, whereas “Open Communication” and “Hospital Management” were identified as areas requiring improvement. Overall, the PT-HSOPSC 2.0 demonstrated robust psychometric properties, confirming its suitability for assessing PSC in Portuguese hospitals.

**Conclusion:**

This study contributes to enhancing PSC assessment in Portuguese healthcare settings, by providing a translated and validated version of the HSOPSC 2.0 adapted to the Portuguese context. Findings support that PT-HSOPSC 2.0 is a reliable and valid tool for evaluating PSC in Portuguese healthcare settings.

## Background

The concept of “safety culture” emerged in the aftermath of the 1988 Chernobyl nuclear disaster and has since been adopted by various industries and organizations to improve safety. In 1999, the Institute of Medicine [IOM] report, To Err is Human, brought this concept to the healthcare sector [1]. This report brought national attention to the prevalence of medical errors in the U.S. healthcare system, identifying them as a leading cause of death and injury. It highlighted the critical need for developing a safety culture in healthcare settings to enhance patient safety and improve care quality. According to the IOM, fostering an environment where adverse events can be reported without assigning blame allows organizations to learn from mistakes. This approach creates opportunities to make improvements and reduce the likelihood of future human and system errors, ultimately improving patient safety [2]. Since then, numerous studies have highlighted the importance of safety culture in improving healthcare safety [1].

According to the Agency for Healthcare Research and Quality [AHRQ], the safety culture within an organization is defined as “ *the product of individual and group values*,* attitudes*,* perceptions*,* competencies*,* and patterns of behavior that determine the commitment to*,* and the style and proficiency of*,* an organization’s health and safety management*.” [3]. Therefore, Patient safety culture [PSC] in healthcare refers to the values, beliefs, norms, and capabilities of individuals and organizations that determine their commitment and actions towards patient safety. Promoting PSC and assessing it are essential for improving patient safety. PSC assessment has multiple uses, including raising awareness of patient safety among professionals; identifying strengths and opportunities for improvement; assessing the current state of PSC; evaluating the impact of actions and initiatives to be implemented in the development of PSC; analyzing the evolution of PSC; and comparing services within healthcare organizations, or even national and international benchmarking between different healthcare organizations [4]. Improved levels of PSC have also been linked to shorter hospital stays, fewer readmissions, and medication errors. This underscores the importance of maintaining a positive PSC to minimize errors and adverse events [5].

The World Health Organization’s [WHO] Global Patient Safety Action Plan 2021–2030 recommends that all countries promote and enhance the culture of patient safety in their healthcare systems. A key component of this effort is “*Strategic Objective 2: Build high-reliability health systems and health organizations that protect patients daily from harm*”. This includes conducting regular safety culture assessments and participating in international benchmarking initiatives. This approach allows healthcare systems to identify their strengths and areas for improvement regarding safety culture. Insights from other countries can be used to enhance patient safety and mitigate adverse events [6].

The WHO Global Patient Safety Report 2024 also emphasizes the importance of cultivating and maintaining a culture within healthcare organizations that fosters respect, openness, and transparency. Such a culture should prioritize learning over blame or retribution. Additionally, the report highlights that a positive safety culture, characterized by trust, shared safety perceptions, and a focus on learning from errors, is essential for effective patient safety management. Ultimately, a strong safety culture is vital for effectively implementing and sustaining patient safety interventions [7].

The most frequently used method for assessing the level of PSC in healthcare organizations is the quantitative approach. This involves administering anonymous surveys that measure various dimensions of PSC [1]. These instruments are considered an efficient strategy and have been the focus of many review studies that compare their general characteristics and examine their psychometric properties [8]. The Hospital Survey on Patient Safety Culture [HSOPSC] of AHRQ is one of the most widely used tools internationally, with excellent psychometric properties and it has been subject to several translations and cultural adaptations [5, 8].

Portugal has shown its dedication to improving patient safety and healthcare quality through its PSC efforts. Legislative documents reflect the government’s commitment to this goal [9, 10]. Previous research by Eiras et al. investigated the validity and reliability of the first version of HSOPSC in Portuguese hospitals. The study provided foundational insights into the PSC landscape. Eiras et al. concluded that the dimensions of PSC present in HSOPSC 1.0 were appropriate for the Portuguese population. The study also highlighted the importance of using hospital culture assessment tools to develop effective strategies and projects for patient safety in Portuguese healthcare organizations [11]. Since then, the Portuguese version of HSOPSC has been used to assess the patient safety culture of Portuguese hospitals, enabling national and international benchmarking and identifying strengths and opportunities for improvement in Portuguese hospitals [12].

In 2019, AHRQ released HSOPSC 2.0, incorporating feedback and suggestions from various countries that have implemented HSOPSC 1.0. AHRQ stated that starting in 2020, the 1.0 benchmarks would no longer be updated and that a direct comparison between the 1.0 and 2.0 surveys would not be possible. They recommended transitioning the research [13].

Considering that it is crucial to continue the international benchmarking of PSC in our Portuguese hospitals in the future, the present study aims to update the Portuguese version of HSOPSC and to determine the psychometric properties of the Portuguese version of HSOPSC 2.0, to continue assessing and characterizing PSC in Portuguese hospitals.

## Materials and methods

### Study design

This is a two-phase study. The first phase involved the translation and cultural adaptation of the HSOPSC 2.0 into the Portuguese language and culture. The second phase of the study is a cross-sectional study and consists of the implementation of the translated version of HSOPSC 2.0, to assess the psychometric characteristics of the survey and to allow the PSC assessment of the participating hospitals.

### Instrument

Among the various validated scales for measuring safety culture, the Hospital Survey on Patient Safety Culture (HSOPSC) is the most widely used instrument internationally. Developed by the United States Agency for Healthcare Research and Quality (AHRQ) in 2004, the HSOPSC underwent a major revision in 2019, resulting in the release of version 2.0.

HSOPSC 2.0 retains the assessment of several key areas of patient safety culture from its predecessor but incorporates significant modifications. While the original HSOPSC 1.0 comprised 51 items and 12 composite measures, the updated HSOPSC 2.0 includes 40 items and evaluates 10 composite measures of patient safety culture. Two dimensions (Overall Perceptions of Patient Safety and Teamwork Across Units) were dropped, and some dimension names were revised to better align with the constructs they measure, enhancing clarity and interpretability.

Each of the 10 composite measures in HSOPSC 2.0 is assessed through 3 to 4 items distributed throughout the survey. Of the 32 items, 13 are negatively worded and were reverse-coded, while 19 are positively worded. Additionally, the survey includes two single-item measures: one assessing the number of patient safety events reported by the respondent and another providing an overall patient safety rating for their service/unit. Furthermore, HSOPSC 2.0 contains six contextual items that collect general demographic and professional information, such as occupational category, service/unit, length of service, and patient contact. Table 1 presents the 10 composite measures assessed in HSOPSC 2.0, along with their corresponding items.

**Table 1:** Composite measures and items of the HSOPSC 2.0

Composite measures (Response options)		Item number	Item
1	Teamwork (Strongly Disagree, Disagree, Neither Agree nor Disagree, Agree, Strongly Agree, Does Not Apply or Don’t Know)	A1	In this unit, we work together as an effective team.
		A8	During busy times, staff in this unit help each other.
		A9r^*^	There is a problem with disrespectful behavior by those working in this unit.
2	Staffing and Work Pace (Strongly Disagree, Disagree, Neither Agree nor Disagree, Agree, Strongly Agree, Does Not Apply or Don’t Know)	A2	In this unit, we have enough staff to handle the workload.
		A3r^*^	Staff in this unit work longer hours than is best for patient care.
		A5r^*^	This unit relies too much on temporary, float, or PRN staff.
		A11r^*^	The work pace in this unit is so rushed that it negatively affects patient safety.
3	Organizational Learning — Continuous Improvement (Strongly Disagree, Disagree, Neither Agree nor Disagree, Agree, Strongly Agree, Does Not Apply or Don’t Know)	A4	This unit regularly reviews work processes to determine if changes are needed to improve patient safety.
		A12	In this unit, changes to improve patient safety are evaluated to see how well they worked.
		A14r^*^	This unit lets the same patient safety problems keep happening.
4	Response to Error (Strongly Disagree, Disagree, Neither Agree nor Disagree, Agree, Strongly Agree, Does Not Apply or Don’t Know)	A6r^*^	In this unit, staff feel like their mistakes are held against them.
		A7r^*^	When an event is reported in this unit, it feels like the person is being written up, not the problem.
		A10	When staff make errors, this unit focuses on learning rather than blaming individuals.
		A13r^*^	In this unit, there is a lack of support for staff involved in patient safety errors.
5	Supervisor, Manager, or Clinical Leader Support for Patient Safety (Strongly Disagree, Disagree, Neither Agree nor Disagree, Agree, Strongly Agree, Does Not Apply or Don’t Know)	B1	My supervisor, manager, or clinical leader seriously considers staff suggestions for improving patient safety.
		B2r^*^	My supervisor, manager, or clinical leader wants us to work faster during busy times, even if it means taking shortcuts.
		B3	My supervisor, manager, or clinical leader takes action to address patient safety concerns that are brought to their attention.
6	Communication About Error (Never, Rarely, Sometimes, Most of the time, Always, Does Not Apply or Don’t Know)	C1	We are informed about errors that happen in this unit.
		C2	When errors happen in this unit, we discuss ways to prevent them from happening again.
		C3	In this unit, we are informed about changes that are made based on event reports.
7	Communication Openness (Never, Rarely, Sometimes, Most of the time, Always, Does Not Apply or Don’t Know)	C4	In this unit, staff speak up if they see something that may negatively affect patient care.
		C5	When staff in this unit see someone with more authority doing something unsafe for patients, they speak up.
		C6	When staff in this unit speak up, those with more authority are open to their patient safety concerns.
		C7r^*^	In this unit, staff are afraid to ask questions when something does not seem right.
8	Reporting Patient Safety Events (Never, Rarely, Sometimes, Most of the time, Always, Does Not Apply or Don’t Know)	D1	When a mistake is caught and corrected before reaching the patient, how often is this reported?
		D2	When a mistake reaches the patient and could have harmed the patient, but did not, how often is this reported?
9	Hospital Management Support for Patient Safety (Strongly Disagree, Disagree, Neither Agree nor Disagree, Agree, Strongly Agree, Does Not Apply or Don’t Know)	F1	The actions of hospital management show that patient safety is a top priority.
		F2	Hospital management provides adequate resources to improve patient safety.
		F3r^*^	Hospital management seems interested in patient safety only after an adverse event happens.
10	Handoffs and Information Exchange (Strongly Disagree, Disagree, Neither Agree nor Disagree, Agree, Strongly Agree, Does Not Apply or Don’t Know)	F4r^*^	When transferring patients from one unit to another, important information is often left out.
		F5r^*^	During shift changes, important patient care information is often left out.
		F6	During shift changes, there is adequate time to exchange all key patient care information.
Number of Events Reported (None, 1 to 2, 3 to 5, 6 to 10, 11 or more)		D3	In the past 12 months, how many patient safety events have you reported?
Patient Safety Rating (Poor, Fair, Good, Very Good, Excellent)		E1	How would you rate your unit/work area on patient safety?

Asterisks (^*^) indicate negatively worded items that were subsequently reverse-coded

The HSOPSC 2.0 exhibits strong internal consistency, with Cronbach’s alpha coefficients ranging from 0.67 to 0.89 across the 10 composite measures, indicating adequate to excellent reliability.

Furthermore, the AHRQ recommends that translated versions of the survey preserve the original number of items and dimensions, ensuring conceptual equivalence and comparability across different linguistic and cultural contexts. No items should be eliminated during the translation process [3].

### Translation

The translation process followed the internationally recommended 4-phase forward-backward translation method to ensure linguistic and cross-cultural equivalence [14]. This process began after obtaining approval for the Portuguese translation and cultural adaptation of the HSOPSC through email correspondence with AHRQ. The steps included forward translation, synthesis of translations, reverse translation, expert panel review, pre-testing, and final version development.

Initially, two independent bilingual translators produced forward translations of the survey into Portuguese. These translations were compared to ensure clarity, cultural relevance, and appropriateness. Discrepancies were discussed and resolved with the translators, resulting in a single reconciled Portuguese version. This version underwent back-translation into English by a native English translator, ensuring that the conceptual meaning aligned with the original survey.

Then, a panel of seven bilingual experts, comprising academics and professionals experienced in quality and patient safety, reviewed the translations. They assessed semantic, cultural, and conceptual equivalence, as well as the overall readability and comprehension of the translated items, response options, and survey instructions. The panel unanimously concluded that the translation effectively captured the intent and meaning of the original questionnaire without requiring overt changes to adapt the survey to the Portuguese context. This process culminated in the final construction of the Portuguese version of the HSOPSC 2.0 [PT-HSOPSC 2.0].

Subsequently, a pre-test was carried out with a sample of 10 hospital employees to assess the comprehensibility and relevance of the questionnaire.

### Settings, sample and data collection

The hospitals included in the sample are part of Portugal’s National Health System. Seven public hospitals participated, geographically distributed throughout the country, representing Portugal’s five regional health administrations. The hospitals ranged in capacity from 85 to 450 beds and employed between 400 and 2,500 professionals.

The data collection method was based on convenience. Hospital selection was based on voluntary participation, while staff selection followed voluntary response sampling, including all staff present during the data collection period, irrespective of their direct interaction with patients. Inclusion criteria required participants to be employed at one of the participating hospitals, to be on duty during the data collection period, and to have the ability to read and understand the Portuguese language. Completion of the questionnaire was considered as implied consent to participate.

A minimum sample of 400 respondents was defined, following the recommendation of at least ten participants per item in instrument validation studies [15]. The survey was conducted electronically from January 18 to February 8, 2023, using SurveyMonkey software after obtaining approval from the Ethics Committees and authorizations from institution’s leaders. An exclusive link was sent to each hospital, guaranteeing participants’ anonymity. All survey items were mandatory except for the open-ended question. During data collection, weekly reminders were sent to increase participation, and updates on response rates were provided to hospital managers.

### Variables

As recommended by AHRQ, the Portuguese version of HSOPSC 2.0 maintains the original structure to ensure comparability of results with the original instrument, keeping the same number of composite measures and items. Consequently, the variables in this study correspond to the composite measures and items derived from the translation process of the survey, as previously shown in Table 1.

### Data analysis

Following AHRQ recommendations, the negative category was created by combining the two lowest response options (Strongly Disagree/Disagree and Never/Rarely) before conducting data analysis. Similarly, the positive category was formed by grouping the two highest response options (Strongly Agree/Agree and Almost Always/Always).

To calculate response frequencies for each survey item, the intermediate scale points (Neither Agree nor Disagree and Sometimes) were classified as a neutral category [3]. Additionally, negatively worded items were reverse-coded to ensure that disagreement with negative statements reflected a positive perception of patient safety culture. This approach followed the guidelines outlined in the HSOPSC 2.0 User’s Guide, which specifies that responses such as “Strongly Disagree” or “Disagree” should be treated as positive scores for negatively worded items [3].

Data analysis was performed using IBM SPSS Statistics v.26 to assess the reliability of the instrument. Survey validity was examined through Exploratory Structural Equation Modeling within a Confirmatory Factor Analysis framework (ESEM-within-CFA), conducted using IBM SPSS AMOS v.26.

Composite reliability analysis was assessed through Cronbach’s alpha which evaluates the internal consistency of items within each composite measure. Cronbach’s alpha values between 0.7 and 0.9 indicate high internal consistency, while values around 0.5 are considered acceptable [16].

Factor reliability was assessed through exploratory factor analysis (EFA) using the *Maximum Likelihood* estimation method and *oblique rotation* (where the axes of the factors are rotated in a manner that allows them to be non-orthogonal, i.e., correlated)^1^. The factor loadings represent the degree of association between each item and its corresponding theoretical factor.

CFA was conducted using the maximum likelihood estimation method. The assumptions for structural analysis were met, including the absence of outliers, multivariate normality (with skewness and kurtosis values close to zero), and absence of multicollinearity (with Variance Inflation Factor [VIF] < 5 and tolerance > 0.1) [16].

To evaluate the model’s adequacy, absolute fit indices were used, indicating the extent to which the proposed model fits the data in relation to a saturated model. Among these, the χ²/df ratio adjusts the chi-square goodness-of-fit statistic based on its degrees of freedom (df). A ratio close to 1 indicates a perfect fit, while values below 3 are considered good, between 3 and 5 are acceptable, and above 5 suggest poor model fit [18].

Relative fit indices compare the estimated model to a baseline model, providing a measure of its relative improvement. The Goodness-of-Fit Index (GFI) assesses absolute fit, with values below 0.90 indicating a poor fit, between 0.90 and 0.95 reflecting a good fit, and above 0.95 suggesting a very good fit. The Normed Fit Index (NFI) evaluates the improvement in model fit relative to the null model, with values of 0.95 or higher indicating an excellent fit [18].

The Comparative Fit Index (CFI) is an incremental fit index, where values of 0.90 or higher denote a good model fit. Similarly, the Tucker-Lewis Index (TLI), also known as the Non-Normed Fit Index (NNFI), indicates a good fit when exceeding 0.90 [18].

Parsimony indices assess the trade-off between model complexity and fit by penalizing excessive parameterization. The Parsimony Comparative Fit Index (PCFI) adjusts the CFI for model parsimony, while the Parsimony Goodness-of-Fit Index (PGFI) applies a similar penalty to the GFI [18].

Finally, the Root Mean Square Error of Approximation (RMSEA) quantifies the discrepancy between the estimated covariance matrix and the population covariance matrix, with values below 0.08 indicating a good fit [18].

Convergent validity refers to the extent to which items within the same composite dimension are related as predicted by the underlying theory. Convergent validity of a factor was assessed based on the Average Variance Extracted (AVE) value, considered adequate when exceeds 0.50, indicating that the factor explains, on average, more than 50% of the variance of its items [17].

Divergent validity assesses whether a factor correlates more strongly with its own theoretical dimension than with other dimensions. The divergent validity of each factor was evaluated using Pearson’s correlation coefficients between the composite dimensions. Correlations were interpreted as weak when |r| < 0.25, moderate when 0.25 ≤ |r| < 0.5, strong when 0.5 ≤ |r| < 0.75, and very strong when |r| ≥ 0.75. Divergent validity is considered established when the correlation between factors is lower than the corresponding AVE value, thus further supporting the presence of adequate convergent validity [17].

A significance level of 5% (α = 0.05) was adopted for all statistical analyses.

## Results

### Sample and socio-demographic characteristics

The study had an overall average response rate of 32%, with a total of 2604 valid surveys. Participation rates varied between hospitals, ranging from 2 to 55%.

The distribution of professional categories among respondents (Table 2) highlighted the predominance of nurses, who comprised 46% of the study cohort. Other clinical staff, such as dietitians, pharmacists, psychologists, social workers, technicians, therapists, or clinical auxiliaries (24.5%), physicians (11.5%), and support staff, such as unit clerks, office staff, informatics, catering, cleaning, maintenance, or infrastructure staff (16.8%) and management staff (1.2%) were also represented.

**Table 2:** Respondent distribution by professional categories (*n* = 2604)

Total number of participants	*n*	%
Physicians	299	11.5
Nurses	1197	46
Other clinical staff	638	24.5
Support staff	438	16.8
Management	32	1.2

n – absolute frequency; % – relative frequency percentage

Regarding the demographic profile of the participants (Table 3), 61% reported working in the same hospital for 11 or more years, while 41% had been in the same work area or unit for at least 11 years. Additionally, 78% of respondents indicated working between 30 and 40 h per week, and 83% reported frequent or direct interaction with patients.

**Table 3:** Demographics characteristics of study respondents (*n* = 2604)

	*n*	%
Time working at that hospital:		
Less than 1 year	88	3
1 to 5 years	588	23
6 to 10 years	333	13
11 or more years	1595	61
Time working in the current unit/work area:		
Less than 1 year	185	7
1 to 5 years	901	35
6 to 10 years	451	17
11 or more years	1067	41
Hours working per week, typically:		
Less than 30 h per week	33	1
30 to 40 h per week	2035	78
More than 40 h per week	536	21
Direct interaction or contact with patients:		
Typically, yes	2156	83
Typically, no	448	17

n – absolute frequency; % – relative frequency percentage

### Reliability analysis

Table 4 presents the composite and factor reliability values for each dimension of the Portuguese version of the HSOPSC 2.0. The results indicate that Cronbach’s alpha coefficients ranged from 0.63 to 0.88. Except for “Staffing and Work Pace” (α = 0.63) and “Handoffs and Information Exchange” (α = 0.67), all other dimensions demonstrated alpha values above 0.70, suggesting good internal consistency.

**Table 4:** PT-HSOPSC 2.0: composite and factor reliability

Composite Measures	Composite reliability (Cronbach’s alpha)		Factor reliability
	PT-HSOPSC 2.0	Original HSOPSC 2.0	PT-HSOPSC 2.0
Teamwork	0.76	0.76	0.85
Staffing and Work Pace	0.63	0.67	0.73
Organizational Learning – Continuous Improvement	0.80	0.76	0.85
Response to Error	0.83	0.83	0.87
Supervisor, Manager, or Clinical Leader Support for Patient Safety	0.82	0.77	0.89
Communication About Error	0.87	0.89	0.93
Communication Openness	0.78	0.83	0.80
Reporting Patient Safety Events	0.88	0.75	0.93
Hospital Management Support for Patient Safety	0.82	0.77	0.89
Handoffs and Information Exchange	0.67	0.72	0.80

A comparative analysis between the original and adapted versions of the instrument reveals a high degree of similarity in internal consistency reliability. “Teamwork” (α = 0.76) and “Response to Error” (α = 0.83) exhibited identical reliability coefficients in both versions. “Communication About Error” showed strong reliability, with alpha values exceeding 0.80 in both versions. While most dimensions demonstrated similar or improved reliability in the Portuguese version, “Communication Openness” and “Handoffs and Information Exchange” presented slightly lower alpha values compared to the original version.

Factor reliability values further support the robustness of the PT-HSOPSC 2.0. The lowest factor loading was observed for “Staffing and Work Pace” (0.73), while the remaining loadings exceeded 0.80, indicating strong construct reliability. The highest factor loadings were found for “Reporting Patient Safety Events” and “Communication About Error” (both 0.93), reinforcing the instrument’s consistency in assessing patient safety culture in Portuguese healthcare settings.

### Construct validity

**Fig. 1:**
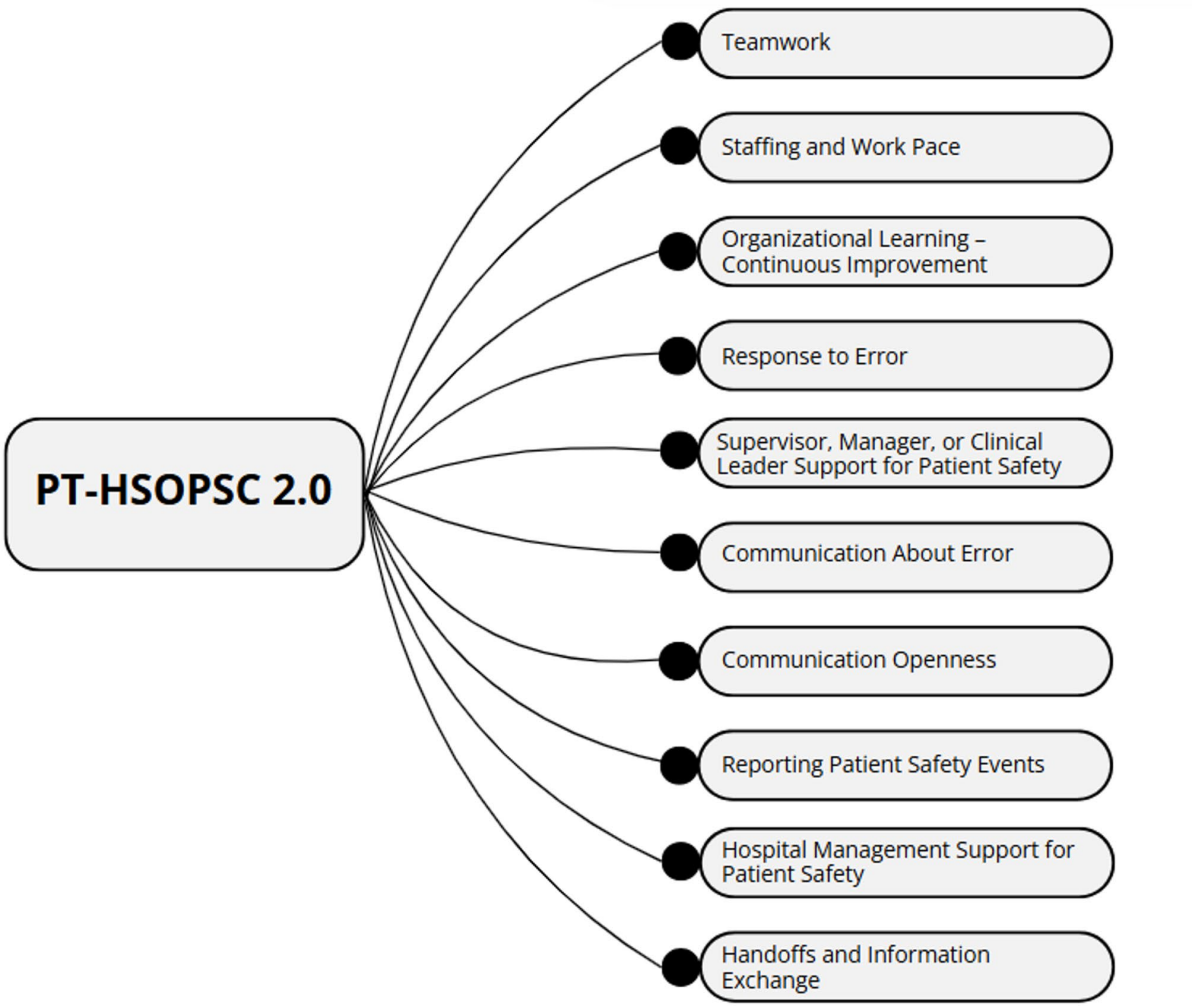
PT-HSOPSC 2.0: confirmatory factor analysis model. Source: Author’s elaboration. Note: The diagram represents the factorial structure resulting from the CFA, with 10 factors arranged vertically (from F1 to F10). The model estimation accounted for the covariance between factors

Figure 1 presents the results of the confirmatory factor analysis (CFA) model. The fit indices obtained were as follows: χ²/df = 4.64, RMSEA = 0.05, CFI = 0.93, GFI = 0.90, TLI = 0.91, PCFI = 0.78, and PGFI = 0.71.

According to the interpretation criteria, the χ^2^/df ratio of 4.64 falls within the acceptable range (between 3 and 5), indicating an adequate model fit. The RMSEA value of 0.05 further supports a good fit, while the CFI (0.93), GFI (0.90), and TLI (0.91) values, all exceeding 0.90, indicate a good model fit. Although the PCFI (0.78) and PGFI (0.71) are slightly lower, they still suggest reasonable parsimony. Overall, these results indicate that the model fits the data satisfactorily [7]. 

Table 5 presents the item loadings from the CFA for each composite dimension of the PT-HSOPSC 2.0. The results indicate that most items have satisfactory factor loadings, with values exceeding 0.40, which is generally considered acceptable for ensuring sufficient factor saturation [16]. Item A5 (“This unit relies too much on temporary workers”), representing the dimension “Staffing and Work Pace” exhibits a lower loading (0.329). Despite this, item A5 was retained in the analysis, as its loading remains within an acceptable range and does not significantly undermine the construct validity of the dimension [16].

**Table 5:** PT-HSOPSC 2.0: item-factor structure and loadings from CFA

Item-Factor Structure			Loadings
A9	<---	Teamwork (F1)	0.623
A8	<---	Teamwork (F1)	0.744
A1	<---	Teamwork (F1)	0.808
A11	<---	Staffing and work pace (F2)	0.732
A5	<---	Staffing and work pace (F2)	0.329
A3	<---	Staffing and work pace (F2)	0.470
A2	<---	Staffing and work pace (F2)	0.661
A14	<---	Organizational learning – continuous improvement (F3)	0.738
A12	<---	Organizational learning – continuous improvement (F3)	0.702
A4	<---	Organizational learning – continuous improvement (F3)	0.718
A13	<---	Response to error (F4)	0.712
A10	<---	Response to error (F4)	0.716
A7	<---	Response to error (F4)	0.673
A6	<---	Response to error (F4)	0.692
B3	<---	Supervisor, Manager, or Clinical Leader Support for Patient Safety (F5)	0.885
B2	<---	Supervisor, Manager, or Clinical Leader Support for Patient Safety (F5)	0.597
B1	<---	Supervisor, Manager, or Clinical Leader Support for Patient Safety (F5)	0.861
C1	<---	Communication About Error (F6)	0.796
C2	<---	Communication About Error (F6)	0.879
C3	<---	Communication About Error (F6)	0.824
C4	<---	Communication Openness (F7)	0.598
C5	<---	Communication Openness (F7)	0.583
C6	<---	Communication Openness (F7)	0.808
C7	<---	Communication Openness (F7)	0.627
D1	<---	Reporting patient safety events (F8)	0.901
D2	<---	Reporting patient safety events (F8)	0.868
F1	<---	Hospital Management Support for Patient Safety (F9)	0.879
F2	<---	Hospital Management Support for Patient Safety (F9)	0.825
F3	<---	Hospital Management Support for Patient Safety (F9)	0.624
F4	<---	Handoffs and information exchange (F10)	0.768
F5	<---	Handoffs and information exchange (F10)	0.823
F6	<---	Handoffs and information exchange (F10)	0.410

For the remaining items, factor loadings range from 0.470 to 0.901, reflecting strong associations between items and their respective dimensions. Items within dimensions such as “Teamwork” [F1], “Organizational Learning – Continuous Improvement” [F3], and “Reporting Patient Safety Events” [F8] exhibit loadings above 0.70, indicating robust item-factor relationships. Overall, the CFA results support the robustness of the factor structure, with most items demonstrating adequate factor loadings, contributing to the construct validity of the PT-HSOPSC 2.0.

### Convergent and divergent validity

Convergent and divergent validity are assessed based on the Average Variance Extracted (AVE) and Pearson’s correlation coefficients, respectively. Table 6 presents the AVE values for each composite measure on the diagonal, indicating the amount of variance explained by each factor, and the Pearson’s correlation coefficients above the diagonal.

**Table 6:** PT-HSOPSC 2.0: convergent and divergent validity

Composite Measures	F1	F2	F3	F4	F5	F6	F7	F8	F9	F10
F1	**0.659**									
F2	0.230	**0.418**								
F3	0.585	0.454	**0.648**							
F4	0.622	0.435	0.877	**0.620**						
F5	0.450	0.304	0.749	0.636	**0.741**					
F6	0.381	0.212	0.664	0.538	0.512	**0.806**				
F7	0.499	0.249	0.680	0.669	0.670	0.715	**0.571**			
F8	0.158	0.104	0.372	0.268	0.247	0.410	0.342	**0.559**		
F9	0.243	0.416	0.501	0.412	0.358	0.281	0.336	0.207	**0.732**	
F10	0.168	0.146	0.209	0.194	0.146	0.114	0.210	0.078	0.108	**0.589**

The values of Average Variance Extracted (AVE) are presented on the diagonal in bold; values below the diagonal represent Pearson’s correlation coefficients

F1 - Teamwork; F2 - Staffing and Work Pace; F3 - Organizational Learning – Continuous Improvement; F4 - Response to Error; F5 - Supervisor, Manager, or Clinical Leader Support for Patient Safety; F6 - Communication About Error; F7 - Communication Openness; F8 - Reporting Patient Safety Events; F9 - Hospital Management Support for Patient Safety; F10 - Handoffs and Information Exchange

The composite measure “Staffing and Work Pace” [F2] presents an AVE value of 0.418, which is below the recommended threshold of 0.50, suggesting that this factor may not fully explain the variance of its items. Despite this, it remains within an acceptable range and is considered to have adequate convergent validity [17]. The remaining factors exhibit AVE values above 0.50, indicating good convergent validity.

The composite measures “Reporting Patient Safety Events” [F8], “Hospital Management Support for Patient Safety” [F9], and “Handoffs and Information Exchange” [F10] demonstrate good divergent validity, as their AVE values are higher than the Pearson’s correlation coefficients with other composite measures.

“Teamwork” [F1] demonstrates moderate to strong correlations with other dimensions (e.g., 0.585 with “Organizational Learning – Continuous Improvement” [F3] and 0.622 with “Response to Error” [F4]), but still exhibits sufficient divergent validity because its correlations are lower than its own AVE value (0.659).

“Staffing and Work Pace” [F2] shows moderate correlations with “Organizational Learning – Continuous Improvement” [F3] (0.454) and “Response to Error” [F4] (0.435), both higher than its AVE value (0.418), indicating weak divergent validity.

“Organizational Learning – Continuous Improvement” [F3] fails to meet the divergent validity criterion, as it exhibits strong correlations (above 0.6) with “Response to Error” [F4] (0.877), “Supervisor, Manager, or Clinical Leader Support for Patient Safety” [F5] (0.749), “Communication About Error” [F6] (0.664), and “Communication Openness” [F7] (0.680).

“Response to Error” [F4] also fails to meet the divergent validity criterion, as it reveals strong correlations (above 0.5) with “Supervisor, Manager, or Clinical Leader Support for Patient Safety” [F5] (0.636), “Communication About Error” [F6] (0.538), and “Communication Openness” [F7] (0.669).

Overall, the results presented in Table 6 indicate that all composite measures demonstrate adequate convergent validity, as evidenced by AVE values exceeding the 0.50 threshold. However, only the composite measures “Reporting Patient Safety Events” [F8], “Hospital Management Support for Patient Safety” [F9], and “Handoffs and Information Exchange” [F10] exhibit clear divergent validity. These measures show that their correlations with other factors are lower than their own AVE values, which is a critical indicator of divergent validity. In contrast, the factors “Teamwork” [F1] and “Staffing and Work Pace” [F2] show moderate divergent validity, as their correlations with other dimensions approach or exceed the AVE values, suggesting some overlap with other constructs.

For the remaining factors — “Organizational Learning – Continuous Improvement” [F3], “Response to Error” [F4], “Supervisor, Manager, or Clinical Leader Support for Patient Safety” [F5], “Communication About Error” [F6], and “Communication Openness” [F7] — stronger correlations between them slightly undermine their divergent validity. These overlaps suggest that these factors may not be sufficiently distinct from one another, which could raise concerns regarding the discriminant validity of the instrument at a more granular level.

### Patient safety culture assessment

In general, the composite measures considered most positive by the majority of healthcare professionals were “Teamwork” (72%); followed by " Supervisor, Manager, or Clinical Leader Support for Patient Safety " (67%); and “Handoffs and Information Exchange” (61%), which represent the strengths of the patient safety culture assessment. On the other hand, opportunities for improvement relate to the composite measure with the lowest percentage of positive responses, such as “Reporting Patient Safety Events” (38%) and “Hospital Management Support for Patient Safety” (39%), as shown in Table 7.

**Table 7:** Patient safety assessment

Composite measures	Average of Positive Responses PT- HSOPSC 2.0 (%)
Teamwork	72
Staffing and Work Pace	45
Organizational Learning—Continuous Improvement	52
Response to Error	45
Supervisor, Manager, or Clinical Leader Support for Patient Safety	67
Communication About Error	47
Communication Openness	58
Reporting Patient Safety Events	38
Hospital Management Support for Patient Safety	39
Handoffs and Information Exchange	61

When professionals were asked about the number of reports made in the last 12 months, it seems that there is no culture of reporting in all hospitals, where 78% of the professionals surveyed said that they had not made any reports in the last year.

The open-ended question allowed participants to share additional insights regarding PSC in their healthcare settings. A qualitative analysis was conducted to identify recurring themes. The most frequently mentioned aspects included the need for improved communication among healthcare professionals, the absence of feedback on incident reporting and concerns about staffing levels. Additionally, there was an emphasis on the importance of continuous training in patient safety and the promotion of a non-punitive culture for error reporting.

## Discussion

The Portuguese version of the Hospital Survey on Patient Safety Culture 2.0 (PT-HSOPSC 2.0) was meticulously translated and culturally adapted, and then rigorously evaluated. Following the methodological process of translation and evaluation of cultural and conceptual semantic equivalence of the survey, which included a panel of experts, it was concluded that all items were relevant and applicable to the Portuguese hospital context and none of the items were removed, in a similar manner to China, Brazil, Turkish, Indonesia, and Malaysia [5, 19–22]. However, item A5 was removed from the Korean version of HSOPSC 2.0 and in the Chilean version both items and composites measures were reduced [23, 24]. Therefore, Portuguese version maintained the original structure of HSOPSC 2.0 to facilitate comparability with the original instrument, emphasizing the importance of maintaining consistency in composite measures and items. Maintaining fidelity to the original structure ensures comparability of results [13].

The data collection process was designed to minimize missing responses by making all survey items mandatory, except for an optional open-ended question. This approach aimed to ensure completeness and consistency in the dataset. As a result, the dataset did not contain missing responses for the items used in confirmatory factor analysis. The inclusion of mandatory responses was an intentional methodological choice to facilitate robust statistical analysis, without the need for imputation or exclusion of cases due to missing data.

Overall, the results indicate that the Portuguese version of HSOPSC 2.0 exhibits good psychometric properties. The instrument demonstrates both composite and factor reliability, while model fit indices support its construct validity. All factors exhibit convergent validity, and some also demonstrate divergent validity. Additionally, Cronbach’s alpha and composite reliability values confirm satisfactory internal consistency.

Notably, the composite measures “Organizational Learning – Continuous Improvement”, “Response to Error”, “Supervisor, Manager, or Clinical Leader Support for Patient Safety”, “Communication About Error”, “Reporting Patient Safety Events”, and “Hospital Management Support for Patient Safety” exhibited strong internal consistency, with Cronbach’s alpha coefficients equalizing or exceeding 0.8. In contrast, “Staffing and Work Pace” and “Handoffs and Information Exchange” showed lower reliability, with values around 0.6, but still reasonable since were above 0.50 [16]. Although they are low, these values indicate a level of internal consistency that does not warrant exclusion of these composite measures [16].

When comparing these findings with those obtained from the original American version, a similar pattern emerges. Specifically, the composite measures “Staffing and Work Pace” and “Handoffs and Information Exchange” exhibited lower reliability in both the Portuguese and American versions, indicating consistency in the instrument’s performance across populations.

The results of the CFA indicate an overall satisfactory model fit for the Portuguese version of HSOPSC 2.0. The key fit indices obtained were χ²/df = 4.64, RMSEA = 0.05, CFI = 0.93, GFI = 0.90, and TLI = 0.91, aligning with established benchmarks for good model fit in psychometric research. The RMSEA value of 0.05 suggests a close approximation between the hypothesized model and the observed data, while the CFI, GFI, and TLI values exceeding 0.90 indicate a strong comparative and absolute fit. However, the parsimony indices (PCFI = 0.78 and PGFI = 0.71) were slightly below the optimal range, suggesting potential areas for model refinement. These findings provide strong evidence supporting the construct validity of the Portuguese version of HSOPSC 2.0, while also indicating that future research could further refine the instrument’s structure to improve its fit indices.

The presence of convergent validity across all composite measures strengthens the internal consistency and reliability of the instrument. However, the lack of clear divergent validity for some composite measures indicates that while these dimensions may be related to their respective theoretical constructs, they also share substantial variance with other dimensions. This suggests that further refinement may be needed to improve the specificity and uniqueness of certain composite measures. These findings underscore the robustness of the instrument while also highlighting areas for enhancement, particularly in ensuring the clear distinction between closely related constructs.

The cross-cultural adaptation of HSOPSC 2.0 to different contexts has revealed challenges related to construct validity and reliability in certain dimensions, as observed in studies from Malaysia and Norway [22, 25]. Thus, while the findings of this study indicate areas that may require adjustments or further investigation, they do not invalidate the instrument’s overall utility. Instead, they highlight the importance and need of ongoing evaluations to enhance the instrument’s applicability and ensure more accurate assessments of patient safety culture across different settings.

The role of PSC in promoting the delivery of safe healthcare has become increasingly prominent and a priority in the Organization for Economic Co-operation and Development [OECD] countries and around the world. Interest in the relationship between the PSC and health outcomes is growing, and there is already some scientific evidence demonstrating the correlation between patient safety culture and improved health outcomes [6]. PSC provides invaluable information about how patient safety is perceived within a healthcare organization. The study of seven Portuguese hospitals revealed that the dimensions with the highest positive percentages were “Teamwork” and “Supervisor, Manager, or Clinical Leader Support for Patient Safety”.

The qualitative findings from the open-ended question provided valuable contextual insights into patient safety culture. Recurring themes included communication challenges, insufficient feedback on incident reporting, and staffing constraints. These issues emphasize that effective teamwork, transparent communication, and adequate human resources are fundamental to ensuring patient safety. Furthermore, the call for continuous training and a non-punitive approach to error reporting underscores the need to strengthen institutional support for safety initiatives. These findings provided valuable contextual information, complementing the quantitative results and reinforcing key areas for improvement in patient safety culture.

### Strengths

This study has several strengths that should be considered. A key strength is the rigorous methodological process applied in the translation and validation of PT-HSOPSC 2.0, following international best practices. The study also facilitates cross-cultural comparisons by maintaining the original structure of the survey, ensuring alignment with international adaptations. Furthermore, the robustness of the psychometric properties confirms the instrument’s validity in the Portuguese hospital context.

### Limitations

However, the study has certain limitations. Although efforts were made to ensure the representativeness by including at least one hospital from each Regional Health Administration of mainland Portugal, the first limitation is the use of convenience sampling. This sampling approach may restrict the generalizability of the findings to all Portuguese healthcare institutions. Another limitation is the response rate obtained in the questionnaire administration, which, while comparable to some international studies, remains suboptimal. Although the response rate in the United States of America was 48%, in the present study reported response rates ranged ranging from 2.29 to 55.98%, with an average of 32.45%. This represents a very low adherence rate, especially considering the recommendations of Pronovost (2005).

### Future investigations

Future research should attempt to achieve higher adherence rates [26] to robustness of findings. Moreover, although the CFA results were generally satisfactory, the slightly lower parsimony indices suggest areas for further refinement of the model’s structure.

The findings of this study also highlight several key areas for future research and practice to advance PSC and improve healthcare environments.

#### Exploring organizational support

Future research could investigate how hospital management can provide stronger, proactive support for patient safety initiatives, moving beyond reactive responses to adverse events. This is particularly relevant given the relatively low percentage of positive responses (39%) regarding hospital management’s support for patient safety identified in this study.

#### Comparative studies

Conducting comparative analyses with countries that have translated and adapted the HSOPSC 2.0, such as China, Brazil, Turkey, Indonesia and Malaysia could provide valuable cross-cultural insights. These studies may help identify similarities and differences in PSC assessment methodologies and outcomes, contributing to the global advancement of patient safety practices.

Others futures research directions can be taken to advance understanding, practices, and interventions related to patient safety culture within healthcare. First, refining and validating PSC assessment tools, such as the HSOPSC, should remain a priority, with the inclusion of complementary items like those addressing Hospital Workplace Safety by AHRQ. This aligns with the dual goals of fostering a healthier work environment for healthcare professionals and improving the quality and safety of care. Additionally, targeted strategies to enhance reporting culture are crucial, as the low percentage of professionals reporting safety events (78% reported no events) underscores the need to encourage and facilitate reporting practices. Research into communication practices, including openness, error communication, and effective handoff protocols, can further provide actionable insights into strengthening PSC. Finally, longitudinal studies that monitor changes in PSC over time are essential to evaluate the long-term impact of interventions and initiatives aimed at improving patient safety.

Together, these insights provide a roadmap for advancing both research and practical efforts in patient safety culture, ensuring continuous improvement in healthcare environments.

## Conclusion

This study provides a comprehensive evaluation of patient safety culture in Portuguese healthcare settings by translating, adapting, and validating the HSOPSC 2.0 survey to the Portuguese context. In doing so, it underscores the significance of accurately assessing PSC within healthcare organizations, particularly in the context of Portuguese hospitals.

The findings from this research offer strong evidence supporting the reliability and validity of the PT-HSOPSC 2.0 as an effective tool for assessing PSC in Portuguese healthcare settings. This validated version is instrumental in advancing the understanding of PSC and fostering improvements in safety practices across the country.

While the PT-HSOPSC 2.0 demonstrates robust psychometric properties, the study also highlights areas where further refinement may be needed, particularly in enhancing the specificity and uniqueness of certain composite measures. These insights provide valuable guidance for refining the tool, ensuring its continued applicability and precision in capturing the nuances of PSC in diverse healthcare environments.

Ultimately, this study contributes significantly to the enhancement of PSC assessment in Portugal, supporting ongoing efforts to improve patient safety practices, guide benchmarking initiatives, and inform quality improvement strategies within Portuguese hospitals. The results not only enrich the existing body of research on PSC but also offer insights for enhancing safety and quality in healthcare delivery.

## Data Availability

The datasets used and/or analyzed during the current study are available from the corresponding author upon reasonable request.

## Footnotes

1 EFA assumptions were thoroughly checked prior to conducting the analysis. Kaiser normalization (eigenvalues greater than 1) was applied to ensure the extraction of meaningful factors. Communalities, defined as the proportion of the total variance of each observed variable explained by the extracted factors, were confirmed to be above the threshold of 0.30. The adequacy of the sample for factor analysis was assessed using the Kaiser-Meyer-Olkin (KMO) measure, with values closer to 1 indicating better suitability of the data for factor analysis and a clearer underlying structure. A KMO value greater than 0.7 is generally considered acceptable for proceeding with factor analysis, while values above 0.8 are considered indicative of good adequacy [17]. Additionally, Bartlett’s test of sphericity yielded a significant result, enabling the rejection of the null hypothesis, which posits that the correlation matrix is an identity matrix. This outcome further supported the appropriateness of conducting the factor analysis [17].
